# Psychometric properties of an Arabic translation of the body appreciation scale (BAS-2) and its short forms (BAS-2SF) in a community sample of Lebanese adults

**DOI:** 10.1186/s40337-023-00885-x

**Published:** 2023-09-18

**Authors:** Feten Fekih-Romdhane, Vanessa Azzi, Diana Malaeb, Abir Sarray El Dine, Sahar Obeid, Souheil Hallit

**Affiliations:** 1 The Tunisian Center of Early Intervention in Psychosis, Department of Psychiatry “Ibn Omrane”, Razi Hospital, 2010 Manouba, Tunisia; 2 Faculty of Medicine of Tunis, Tunis El Manar University, Tunis, Tunisia; 3 School of Medicine and Medical Sciences, Holy Spirit University of Kaslik, P.O. Box 446, Jounieh, Lebanon; 4 College of Pharmacy, Gulf Medical University, Ajman, United Arab Emirates; 5 Department of Biomedical Sciences, School of Arts and Sciences, Lebanese International University, Beirut, Lebanon; 6 School of Arts and Sciences, Social and Education Sciences Department, Lebanese American University, Jbeil, Lebanon; 7 Psychology Department, College of Humanities, Effat University, Jeddah, Saudi Arabia; 8 Applied Science Research Center, Applied Science Private University, Amman, Jordan; 9 Research Department, Psychiatric Hospital of the Cross, Jal Eddib, Lebanon

**Keywords:** BAS-2, BAS-2SF, Body appreciation, Psychometric properties, Arabic

## Abstract

**Objectives:**

The present study sought to examine the factor structure, reliability, validity and gender invariance of the Arabic Body Appreciation Scale (BAS-2), and its two short forms (the 3-item and 2-item BAS-2SF) among community Lebanese Arabic-speaking adults.

**Methods:**

We carried-out an online cross-sectional survey. The 10-item BAS-2, the functionality appreciation scale and the Eating Attitudes Test-26 were completed by 826 Lebanese community adults (57.9% females, aged 25.42 ± 8.44 years).

**Results:**

The Arabic 10-item, 3-item and 2-item BAS-2 converged on a one-factor solution, and demonstrated high internal consistency (McDonald’s ω value of .92, .88, and .86 respectively). All indices suggested that configural, metric, and scalar invariance was supported across gender. The 3-item and 2-item BAS-2SF were highly correlated with the original BAS-2 (r > 0.9). Higher 10-item, 3-item and 2-item BAS-2 scores correlated significantly and strongly with more positive eating attitudes and greater functionality appreciation, and higher 10-item BAS-2 scores correlated positively and weakly with BMI, supporting convergent validity.

**Conclusion:**

The present work contributes to the literature by providing a psychometrically sound Arabic-translation of the BAS-2 and short-forms, benefitting both researchers and clinicians.

**Supplementary Information:**

The online version contains supplementary material available at 10.1186/s40337-023-00885-x.

## Abstract (plain language summary)

Research on body image traditionally has focused on describing and predicting negative body image such as body dissatisfaction, body shame, and body preoccupation, with less focus on identifying, predicting, and promoting adaptive body attitude. Recently, however, the study of positive body image has gained considerable motion, and body appreciation has been the central organizing variable within these investigations. Body appreciation has been defined as accepting, holding favourable opinions toward, and respecting the body, while also rejecting media-promoted appearance ideals as the only form of human beauty. The Body Appreciation Scale has been utilized by researchers to understand features, correlates, and potential outcomes of positive body image. This scale and its short forms are not yet validated in Arabic. To that end, we aimed to translate and validate the Arabic version of the Body Appreciation Scale (BAS-2), and its two short forms (the 3-item and 2-item BAS-2SF) in the present study, which would in turn facilitate improved research and clinical practices related to body appreciation in Arabic-speaking nations. The present findings provide support for the psychometric properties of the Body Appreciation scales in Arabic-speaking adults in Lebanon.

**Supplementary Information:**

The online version contains supplementary material available at 10.1186/s40337-023-00885-x.

## Introduction

Body image can be defined as individual’s perceptions, feelings, and thoughts about their own physical appearance [1], and encompasses both negative (maladaptive) and positive (adaptive) experiences. While research has traditionally focused on negative body image (e.g., body dissatisfaction, body preoccupation, body shame) [2–4], recent studies have increasingly uncovered the protective and adaptive role of positive aspects of body image in a range of physical and psychological health indicators [5–8]. One key positive body image construct that has received considerable attention is body appreciation, which refers to “accepting, holding favourable opinions toward, and respecting the body, while also rejecting media-promoted appearance ideals as the only form of human beauty” ([8], p. 53). Beyond gratitude toward the body, this concept includes acceptance of features, health, and functions of the body [9]. When conceptualizing the construct, Avalos, Tylka, and Wood-Barcalow [2] initially developed the Body Appreciation Scale (BAS), a 13-item one-factor measure that further supported body appreciation as a distinct and useful construct in the body image field [8]. Later, some shortcomings of the BAS have been identified such as an unsupported unidimensionality [10] or some item phrasing problems (e.g., items containing terms rather related to a negative body evaluation, referring to an ignorance of the appearance, or being gender-specific); which motivated its revision by Tylka and Wood-Barcalow [11] to a new 10-item version (BAS-2).

Since its original validation in 2015, the BAS-2 has shown its strong psychometric quality within various population-based samples of varying characteristics, including adolescents [12–14], students [15, 16], adults [17, 18], older adults [19], and sexual minorities [20]. The BAS-2 has also been validated in several languages and countries around the world, including Greek [21], Dutch [16], Polish [22], Portuguese [12], Mexican [14], Spanish [23, 24], French [15], Swedish [25], Romanian [26], Chinese [27, 28], Japanese [29], Malay [30], Persian [31], Lithuanian [32], and Arabic [33]. The examination of the BAS-2 psychometric properties in these diverse countries consistently showed a one-dimensional structure, good internal consistency and validity [10], as well as its invariance across gender and country [18]. More recently, on the basis of the questionnaire BAS-2, its short forms (BAS-2SF) consisting of 2 items and 3 items were developed [34]. One item is shared between the two short versions (i.e., “I feel love for my body”), while the rest of the items are different. These versions yielded good psychometric properties, and were therefore recommended by authors for use instead of the full-length scale [34].

### The Arab context

Positive body image concepts, including behavioural expressions toward the body, have been suggested to depend on cultural norms and values, and therefore to express differently across societies, cultures and religions [35–38]. For this reason, Tiggemann [36] encouraged researchers to further investigate the BAS-2 factorial validity in different cultural contexts. The particular context that we are interested in here is the Arab background, which has its unique historical and cultural patterns. Indeed, throughout history, and to some extent currently, Arab people have often had views of body proportions ideals and beauty definitions that diverge from Western body standards [39, 40]. Conversely, research also found that the younger generations tended to become increasingly preoccupied with Western ideal standards of body size and shape as a consequence of modernization and Westernization in the Arab societies [41]. However, very limited research paid specific attention to positive body image concerns in the Arabic-speaking communities and contexts. A recent literature review by Melisse et al. [41] could only identify a few studies investigating body dissatisfaction (e.g. [42–44]), while no studies have been reported on body appreciation or other positive body image constructs in the Arab world. Research on positive body image in the Arab context is likely still hampered by a lack of psychometrically valid assessment measures [41]. It has been only recently that an Arabic version of the BAS-2 has been made available [33]. This Arabic version originated in a relatively small sample of Emiratis female students, demonstrated one-dimensional factor structure, as well as adequate internal consistency and convergent validity [33].

### The present study

In this paper, we propose to test the psychometric properties of an Arabic translation of the BAS-2 and its short forms (the 2-item BAS-2SF and the 3-item BAS-2SF) in a sample of community adults within an Arabic-speaking context, Lebanon. Even though an Arabic version of the scale exists, its validation was limited in certain ways. First, authors relied on a college student sample, which implies that findings may not be generalizable to the wider community. Second, only women have been involved in this validation study, which did not allow for comparisons across genders and for gender invariance to be ascertained. This represents a major gap, given that invariance across women and men of BAS-2 scores yielded mixed findings in previous validations across countries (fully supported in some studies [11, 23, 45], whereas not supported in others [26]). For these reasons, we believe it is useful to extend the validation of the Arabic BAS-2 to include a diverse sample of Arabic-speaking community adult men and women with large variability in demographic features (such as age and educational level). Third, the Arabic version available of the BAS-2 has been validated in the United Arab Emirates, a Gulf Muslim politically and economically stable country. However, while Arab countries share similar social, religious and cultural characteristics [46, 47], diversities do also exist. Therefore, we suggest that the validation of the BAS-2 in an additional Arab country of different religious, cultural, and economic background (i.e., Lebanon; a non-Gulf multireligious and politically/economically unstable country) will enhance its cross-cultural validity. Fourth, providing Arabic psychometrically sound short forms of the BAS-2 can have substantial advantages as they require less time to complete with reduced respondent burden and costs for data collection; which would be of great value in the low- middle income Arab countries.

To this end, we aimed through the present study to examine the factor structure, reliability, validity and gender invariance of the Arabic BAS-2, 2-item BAS-2SF and 3-item BAS-2SF among community Lebanese Arabic-speaking adults. We hypothesized that: (1) the Arabic full-length and short forms of the BAS-2 would have a good internal consistency; (2) our analyses would endorse a good fit of a unidimensional factor structure and show measurement invariance across gender of all three forms of the scale; (3) an examination of the correlations between BAS-2 and BAS-2SF scores, disordered eating attitudes, functionality appreciation, and BMI would provide evidence of convergent validity; and (4) both Arabic BAS-2SF versions’ scores would strongly correlate with the BAS-2 total scores, suggesting that the two short forms evaluate the same body appreciation construct.

## Methods

### Participants

A total of 826 Lebanese citizens and residents enrolled in this study with a mean age of 25.42 years (*SD* = 8.44) and 57.9% females. Other characteristics are displayed in Table 1.

**Table 1:** Sociodemographic characteristics of the participants

Variable	Total sample (N = 826)	Sample 1 (N = 411)	Sample 2 (N = 415)
*Sex*			
Male	348 (42.1%)	182 (44.3%)	166 (40.0%)
Female	478 (57.9%)	229 (55.7%)	249 (60.0%)
*Education*			
Primary	24 (2.9%)	14 (3.4%)	10 (2.4%)
Complementary	48 (5.8%)	30 (7.3%)	18 (4.3%)
Secondary	123 (14.9%)	63 (15.3%)	60 (14.5%)
University	631 (76.4%)	304 (74.0%)	327 (78.8%)

	Mean ± SD		
Age (in years)	25.42 ± 8.44 (min = 18; max = 75)	25.86 ± 9.18 (min = 18; max = 75)	24.97 ± 7.63 (min = 18; max = 64)
Body Mass Index (kg/m^2^)	23.81 ± 4.83 (min = 10.20; max 44.98)	23.82 ± 5.00 (min = 10.20; max = 43.03)	23.80 ± 4.67 (min = 13.84; max = 44.98)

### Measures

#### Body appreciation scale- 2

This 10-item instrument assesses acceptance of one’s body, respect and care for one’s body, and protection of one’s body from unrealistic beauty standards (BAS-2 [11]). Items are rated on a 5-point scale (*never* to *always*). Higher scores on this scale reflect greater body appreciation. Following international guidelines [48], the scale was translated from English into Arabic by a bilingual translator, whose native language is Arabic and who is fluent in English. Next, an expert committee formed by healthcare professionals and a linguistics expert verified the translated Arabic version of the scale. Subsequently, the Arabic version of the scale was back-translated into English by an independent translator who is fluent in Arabic and English. The back-translated measure was returned to the expert committee, who compared both translations and aimed to resolve any inconsistencies between versions. A pilot study was done on 20 persons to make sure all questions are clear [49]. In the present study, the Cronbach’s alpha was 0.96. The BAS-2 items in English are reported in Table 1 and the items in Arabic are reported in Additional file 1: Appendix 1.

#### Functionality appreciation scale (FAS)

The FAS was used to assess how respondents appreciate, respect, and honour their own bodies for what it is capable of doing. The scale is a unidimensional scale, composed of seven items (e.g., “I respect my body for the functions that it performs.) rated on a 5-point scale (*strongly disagree* to *strongly agree*) [50]. Higher scores indicate higher body functionality. The Arabic validated version of the FAS was used in this study [51], which demonstrated good composite reliability, adequate patterns of convergent and criterion-related validity, with 7 items loading into a single factor. In the present sample, the Cronbach’s alpha was 0.95.

#### Eating attitude test-7 items

Participants were asked to complete the Eating Attitudes Test-7 (EAT-7 [52];), which is a shortened version of the Eating Attitude Test-26 (EAT-26 [53]) validated in Arabic [54]. This is a one-factor, seven-item measure of symptoms and concerns characteristic of eating disorders. The Arabic EAT-7 showed excellent reliability and strong evidence of divergent validity [52]. All items are rated on a 6-point scale, ranging from 0 (*never*) to 6 (*always*). The scale is unidimensional, with higher total scores reflecting greater disordered eating attitudes. In the present study, the Cronbach’s alpha was 0.90.

#### Demographics

Participants were asked to provide their demographic details consisting of age, sex, and education level.

#### Body mass index (BMI)

Self-reported data about height and weight were used to compute BMI in kg/m^2^.

### Procedures

Ethics approval for this study was obtained from the Psychiatric Hospital of the Cross ethics committee (approval code: HPC-038-2021). All data were collected via a Google Form link, between December 2021 and April 2022. The project was advertised on social media and included an estimated duration. Inclusion criteria for participation included being of a resident and citizen of Lebanon of adult age. Internet protocol (IP) addresses were examined to ensure that no participant took the survey more than once. After providing digital informed consent, participants were asked to complete the instruments described above, which were presented in a pre-randomised order to control for order effects. The survey was anonymous and participants completed the survey voluntarily and without remuneration.

### Analytic strategy

#### Data treatment

There were no missing responses in the dataset. To examine the factor structure of the BAS-2, we conducted a Confirmatory Factor Analysis (CFA) on the total sample using the SPSS AMOS v.29 software. A previous study suggested that the minimum sample size to conduct a confirmatory factor analysis ranges from 3 to 20 times the number of the scale’s variables [55]. Therefore, we assumed a minimum sample of 200 participants needed to have enough statistical power based on a ratio of 20 participants per one item of the scale, which was exceeded in our sample. Our intention was to test the original model of BAS-2 scores (i.e., a unidimensional model; [11]) and, if divergent, any models extracted from our BAS-2. Parameter estimates were obtained using the maximum likelihood method and fit indices. To check if the model was adequate, several fit indices were calculated: the normed model chi-square (χ^2^/df), the Steiger-Lind root mean square error of approximation (RMSEA), standardized root mean square residual (SRMR), the Tucker-Lewis Index (TLI) and the comparative fit index (CFI). Values ≤ 5 for χ^2^/df, ≤ 0.05 for SRMR, ≤ 0.08 for RMSEA, and 0.90 for CFI and TLI indicate good fit of the model to the data [56]. Additionally, evidence of convergent validity was assessed in this subsample using the Fornell-Larcker criterion, with average variance extracted (AVE) values of ≥ 0.50 considered adequate [57]. Multivariate normality was not verified at first (Bollen-Stine bootstrap p = 0.002); therefore we performed non-parametric bootstrapping procedure (available in AMOS).

To explore the factor structure of BAS-2-SF (2 and 3 items), we computed a principal-axis EFA with the first split-half subsample using the FACTOR software [58]. We verified all requirements related to item-communality (Worthington and Whittaker, 2006), average item correlations, and item-total correlations [59]. The Kaiser–Meyer–Olkin (KMO) measure of sampling adequacy (which should ideally be ≥ 0.80) and Bartlett’s test of sphericity (which should be significant) ensured the adequacy of our sample [60]. The procedure followed for determining the number of dimensions was the Parallel Analysis (PA) [61], using the Pearson correlation matrix. Weighted Root Mean Square Residual (WRMR) were also calculated to assess the model fit (values < 1 have been recommended to represent good fit [62]. Item retention was based on the recommendation that items with “fair” loadings and above (i.e., ≥ 0.33) [63].

#### Gender invariance

To examine gender invariance of BAS-2 scores, we conducted multi-group CFA [64]. Measurement invariance was assessed at the configural, metric, and scalar levels [65]. ΔCFI ≤ 0.010 and ΔRMSEA ≤ 0.015 or ΔSRMR ≤ 0.010 were considered as evidence of invariance [64, 66]. We aimed to test for gender differences on latent BAS-2 scores using an independent-samples *t*-test only if scalar or partial scalar invariance were established.

#### Reliability and validity testing

Composite reliability in both subsamples was assessed using McDonald’s ω and Cronbach’s α, with values greater than 0.70 reflecting adequate composite reliability. Normality was verified since the skewness (= − 0.511) and kurtosis (= − 0.354) values for the total score varied between − 1 and + 1 [67]. To assess concurrent validity, we examined bivariate correlations between BAS-2 scores and functionality appreciation and eating attitudes scores using the Pearson test. Based on Cohen (1992) [68], values ≤ 0.10 were considered weak, ~ 0.30 were considered moderate, and ~ 0.50 were considered strong correlations.

## Results

### Confirmatory factor analysis

CFA indicated that fit of the one-factor model of BAS-2 scores was acceptable: χ^2^/df = 177.32/35 = 5.07, RMSEA = 0.070 (90% CI 0.060, 0.081), SRMR = 0.018, CFI = 0.982, TLI = 0.976. The standardised estimates of factor loadings were all adequate (see Table 2). The convergent validity for this model was adequate, as AVE = 0.71. Composite reliability of scores was excellent in women (ω = 0.95/α = 0.95), men (ω = 0.97/α = 0.97), and the total sample (ω = 0.96/α = 0.96).

**Table 2:** Standardised estimates of factor loadings from the confirmatory factor analysis (CFA) in the total sample

	CFA		
Item	women	Men	Total
1. I respect my body	.80	.87	.84
2. I feel good about my body	.82	.90	.85
3. I feel that my body has at least some good qualities	.78	.83	.81
4. I take a positive attitude towards my body	.87	.88	.88
5. I am attentive to my body’s needs	.77	.83	.79
6. I feel love for my body	.86	.85	.86
7. I appreciate the different and unique characteristics of my body	.83	.88	.86
8. My behaviour reveals my positive attitude toward my body; for example, I hold my head high and smile	.83	.89	.86
9. I am comfortable in my body	.85	.86	.86
10. I feel like I am beautiful even if I am different from media images of attractive people (e.g., models, actresses/actors)	.79	.85	.83

### Gender invariance

As reported in Table 3, all indices suggested that configural, metric, and scalar invariance was supported across gender. Given these results, we computed an independent-samples *t*-test to examine gender differences in BAS-2 scores. The results showed that a higher mean BAS-2 score was seen in women (*M* = 38.11, *SD* = 9.06) compared to men (*M* = 34.54, *SD* = 10.09), *t*(824) = -5.337, *p* < 0.001, *d* = 0.372.

**Table 3:** Measurement invariance across gender in the total sample

Model	χ^2^	*df*	CFI	RMSEA	SRMR	Model Comparison	Δχ^2^	ΔCFI	ΔRMSEA	ΔSRMR	Δ*df*	*p*
Configural	240.28	70	.978	.054	.019							
Metric	261.04	79	.976	.053	.021	Configural vs metric	20.76	.002	.001	.002	9	.013
Scalar	334.26	88	.968	.058	.021	Metric vs scalar	73.22	.008	.005	< .001	9	< .001

CFI = Comparative fit index; RMSEA = Steiger-Lind root mean square error of approximation; SRMR = Standardised root mean square residual

### Validation of the 2- and 3-item BAS-2SF

The 3 items (items 1, 6 and 7) and the 2 items (items 6 and 9) converged on a one-factor solution, with high loadings in the total sample and in men and women separately. Plus, the reliability values were very good (> 0.8) (Table 4).

**Table 4:** Items of the BAS-2 in English and factor loadings derived from the exploratory factor analyses (EFA) in the total sample, women and men

	EFA (3-item BAS-2SF)			EFA (2-item BAS-2SF)		
Item	Women	Men	Total	Women	Men	Total
1	.88	.90	.89			
6	.89	.90	.90	.94	.94	.94
7	.89	.92	.91			
9				.94	.94	.94
McDonald’s omega	.86	.89	.88	.86	.86	.86
Percentage of variance explained	78.36	82.08	80.68	87.39	87.46	87.62

*The 3-item BAS-2SF*: Total Sample: KMO = 0.743; Bartlett’s X2 = 1317.44; df = 3; p < 0.001)

Men: KMO = 0.744; Bartlett’s X2 = 601.70; df = 3; p < 0.001)

Women: KMO = 0.736; Bartlett’s X2 = 665.07; df = 3; p < 0.001)

*The 2–item BAS-2SF*: Total sample: KMO = 0.500; Bartlett’s X2 = 687.82; df = 1; p < 0.001)

Men: KMO = 0.500; Bartlett’s X2 = 284.61; df = 1; p < 0.001)

Women: KMO = 0.500; Bartlett’s X2 = 389.63; df = 1; p < 0.001)

### Convergent and criterion-related validity

To assess the validity of the three BAS-2 scores, we examined bivariate correlations with all other measures included in the present study using the total sample. As can be seen in Table 5, the three BAS-2 scores were significantly and positively correlated with higher functionality appreciation, lower eating attitudes scores (more appropriate eating) and lower BMI (according to the 10- and 2-item BAS-2 scores only). Older age was significantly associated with lower body appreciation (according to the 10- and 3-item BAS-2 scores only).

**Table 5:** Correlations of BAS-2, 3item BAS-2SF and 2–item BAS-2SF with the other measures on the total sample

	10-item BAS-2	3-item BAS-2SF	2-item BAS-2SF
BAS-2	1		
3-item BAS-2SF	.97***	1	
2-item BAS-2SF	.93***	.91***	1
Functionality appreciation	.75***	.76***	.66***
Eating attitudes	− .44***	− .44***	− .37***
Age	− .08*	− .09*	− .06
Body Mass Index	− .07*	− .06	− .12**

*p < 0.05; **p < 0.01; ***p < .001

## Discussion

Despite their peculiarities, Arabic-speaking people have long been neglected in the body image research literature due to a lack of valid and adapted screening tools [41]. While the BAS-2 has been recently validated into the Arabic language [33], a number of gaps remained to be addressed. In this study, we sought to test the factor structure and psychometric properties of the Arabic translation of the BAS-2, and its short forms (the 2-item BAS-2SF and the 3-item BAS-2SF) in a sample of Lebanese Arabic-speaking adults. Our findings provide greater support to the psychometric evidence of the 10-item, 3-item and 2-item BAS-2, preliminarily suggesting the usefulness and feasibility of these three Arabic forms in assessing the body appreciation construct in Arabic-speaking people, at least in the Lebanese context. We believe that the present work would benefit researchers and clinicians by attempting to fill the gaps of the former Arabic validation; and most importantly, contribute to the literature by providing the Arabic short forms of the scale.

Using EFA and CFA, our findings demonstrated that the unidimensional factor structure of the three Arabic forms had a good fit to the data in both genders. This is in agreement with the original English validation of the 10-item BAS-2, where all 10 items loaded onto one factor in both men and women respondents [11]. This is also consistent with previous studies from different countries that demonstrated the goodness-of-fit indices for the original single-factor structure of the BAS-2 (e.g., [11, 12, 15, 16, 22, 23, 28, 29, 31]) as well as of the 3-item and 2-item BAS-2SF [34]. The present findings showed that higher 10-item, 3-item and 2-item BAS-2 scores correlated significantly and strongly with more positive eating attitudes and greater functionality appreciation, and higher 10-item BAS-2 scores correlated positively and weakly with BMI; thus supporting convergent validity of all three forms. Previous research documented similar associations of the BAS-2 with other measures of body image and disordered eating constructs (e.g., disordered eating, body dissatisfaction, appearance evaluation) [2, 10, 69, 70]. In addition, our analyses showed high correlations between the original BAS-2 and the two short-forms, suggesting that the retained two and three items in the shortened versions are informative and relevant to assess the body appreciation construct. We also agree with Tylka et al. suggestion that the Arabic 3-item BAS-2SF is recommended for use when aiming at examining the latent factor structure of the measure, or investigating specific groups (e.g., people with disability, illness, immobility, or pain) [34].

All three Arabic forms of the BAS-2 also evidenced internal consistency and invariance by gender at the metric, configural, and scalar levels. This is in line with findings from the original BAS-2 and BAS-SF validation studies [11, 34], as well as other prior studies across various linguistic and cultural contexts (e.g., [15, 18, 19, 45]). Furthermore, contrary to expectations based on body image literature [71], we found that Arabic-speaking women in our sample displayed higher body appreciation levels compared to men. While female gender has consistently been identified as a risk factor for body-image dissatisfaction and weight-control behaviours [72–75] and male gender has consistently been found to relate to more favourable body appreciation across cultures [76, 77], research emerging from Arab countries found reversed gender patterns. For instance, Alharballeh and Dodeen [78] found that Emiratis male students were more ashamed and dissatisfied with their physical appearance, and more avoidant of situations in which others can see their bodies, as compared to their female counterparts. There is evidence that Arabs exhibit specific culture-related preferences for “heavier”, “curvier” female bodies [39, 40]. This is plausible particularly knowing that physical activities are culturally less common in Arab women as compared to men [78]. This may result in women being less prone to be involved in intensive physical exercising, more likely to consider themselves normal-weight and accept, in turn, their bodies’ size and shape. These data along with our findings highlight that both males and females face social and cultural pressures of body ideals, though with some gender-related specificities [79], and that men also have body image concerns to a similar extent or even more than women [80–82]; which calls for additional focus on body image issues research involving male samples.

### Study limitations

Our findings have to be carefully interpreted bearing in mind certain limitations. The test–retest reliability of the BAS-2 and BAS-2SF was not examined in the context of the present study, precluding any conclusions regarding scores stability across time. In addition, while we provide support for the validity of these Arabic versions in a community sample of Lebanese adults, future research need to assert their psychometric robustness in clinical samples, and samples from Arab countries others than Lebanon. Cross-cultural research need also to ascertain the invariance of these measures across Arab countries and communities. Another limitation is the self-report nature of all measures used in our study that might compromise the accuracy of responses due to social desirability bias.

## Conclusion

While one's relationship with body image is constantly evolving and changing in the Arab world as a result of the globalization process, research in this field has not followed at the same pace. One major and unique contribution of the present study was the examination of gender invariance and comparison of the body appreciation construct across genders in an Arab context. Another contribution was making shorter forms of the BAS-2 available for Arabic-speaking populations. Due to their brevity and good psychometric properties, the Arabic 3-item and 2-item BAS-2SF are advantageous for assessing body appreciation in Arab clinical and research settings, especially when long questionnaires at multiple time points are needed (e.g., repeated clinical monitoring, longitudinal studies). We thus hope that providing these valid, simple, easy-to-use and low-cost tools will be of substantial benefit to clinicians, and will stimulate more research in this area in the Arab context. Such research may inform strategies aiming at promoting the appreciation and respect of one’s body in Arabic-speaking communities worldwide.

### Supplementary Information

**Additional file 1.** Body Appreciation Scale-2.

## Data Availability

All data generated or analyzed during this study are not publicly available due the restrictions by the ethics committee (data are owned by the Psychiatric Hospital of the Cross). The dataset supporting the conclusions is available upon request to Ms. Rana Nader (rnader@naderlawoffice.com), a member of the ethics committee at the Psychiatric Hospital of the Cross.
