# Supplementary material for: Psychometric properties of an Arabic translation of the body appreciation scale (BAS-2) and its short forms (BAS-2SF) in a community sample of Lebanese adults
Source: J Eat Disord. 2023 Sep 18;11:160. doi: 10.1186/s40337-023-00885-x (PMC10506330; doi:10.1186/s40337-023-00885-x)
Supplement: Supplementary file 1 — Additional file 1. Body Appreciation Scale-2. [file 40337_2023_885_MOESM1_ESM.docx]

|  | **أبداً = 1** | **نادراً = 2** | **في بعض الأحيان = 3** | **غالبًا = 4** | **دائماً = 5** |
| --- | --- | --- | --- | --- | --- |
| أحترم جسدي |  |  |  |  |  |
| أشعر بالرضا عن جسدي |  |  |  |  |  |
| أشعر أن جسدي لديه على الأقل بعض الصفات الجيدة |  |  |  |  |  |
| أتّخذ موقفاً إيجابياً تجاه جسدي |  |  |  |  |  |
| أنا منتبه/ منتبهة لاحتياجات جسدي. |  |  |  |  |  |
| أشعر بالحب تجاه جسدي. |  |  |  |  |  |
| أنا أقدّر خصائص جسدي المختلفة والفريدة من نوعها. |  |  |  |  |  |
| سلوكي يكشف عن موقفي الإيجابي تجاه جسدي. على سبيل المثال، أرفع رأسي عالياً وأبتسم. |  |  |  |  |  |
| أنا مرتاح في جسدي. |  |  |  |  |  |
| أشعر أنني جميل/ جميلة حتى لو كنت مختلفًا/ مختلفةً عن صور وسائل الاعلام للأشخاص الجذابين (على سبيل المثال عارضات الأزياء، الممثّلين / الممثّلات) |  |  |  |  |  |

**Body Appreciation Scale-2**
